# Bleeding Risk during Treatment of Acute Thrombotic Events with Subcutaneous LMWH Compared to Intravenous Unfractionated Heparin; A Systematic Review

**DOI:** 10.1371/journal.pone.0044553

**Published:** 2012-09-11

**Authors:** Giorgio Costantino, Elisa Ceriani, Anna Maria Rusconi, Gian Marco Podda, Nicola Montano, Piergiorgio Duca, Marco Cattaneo, Giovanni Casazza

**Affiliations:** 1 Unità Operativa di Medicina Interna II, Dipartimento di Scienze Cliniche “L. Sacco”, Ospedale L. Sacco, Università degli Studi di Milano, Milan, Italy; 2 Dipartimento di Scienze Cliniche “L. Sacco”, Università degli Studi di Milano, Milan, Italy; 3 Unità Operativa di Medicina III, Ospedale San Paolo, Dipartimento di Medicina, Chirurgia e Odonotoiatria, Università degli Studi di Milano, Milan, Italy; Sapienza University of Rome, Italy

## Abstract

**Background:**

Low Molecular Weight Heparins (LMWH) are at least as effective antithrombotic drugs as Unfractionated Heparin (UFH). However, it is still unclear whether the safety profiles of LMWH and UFH differ. We performed a systematic review to compare the bleeding risk of fixed dose subcutaneous LMWH and adjusted dose UFH for treatment of venous thromboembolism (VTE) or acute coronary syndromes (ACS). Major bleeding was the primary end point.

**Methods:**

Electronic databases (MEDLINE, EMBASE, and the Cochrane Library) were searched up to May 2010 with no language restrictions. Randomized controlled trials in which subcutaneous LMWH were compared to intravenous UFH for the treatment of acute thrombotic events were selected. Two reviewers independently screened studies and extracted data on study design, study quality, incidence of major bleeding, patients’ characteristics, type, dose and number of daily administrations of LMWH, co-treatments, study end points and efficacy outcome. Pooled odds ratios (OR) and 95% confidence intervals (CI) were calculated using the random effects model.

**Results:**

Twenty-seven studies were included. A total of 14,002 patients received UFH and 14,635 patients LMWH. Overall, no difference in major bleeding was observed between LMWH patients and UFH (OR = 0.79, 95% CI 0.60–1.04). In patients with VTE LMWH appeared safer than UFH, (OR = 0.68, 95% CI 0.47–1.00).

**Conclusion:**

The results of our systematic review suggest that the use of LMWH in the treatment of VTE might be associated with a reduction in major bleeding compared with UFH. The choice of which heparin to use to minimize bleeding risk must be based on the single patient, taking into account the bleeding profile of different heparins in different settings.

## Introduction

In daily clinical practice, low molecular weight heparins (LMWH) and unfractionated heparin (UFH) are the most commonly prescribed anticoagulant drugs for the treatment of acute thrombotic conditions, such as venous thromboembolism (VTE) and acute coronary syndromes (ACS).

LMWH have some advantages over UFH, including higher bioavailability and a more predictable anticoagulant effect. These properties allow the use of LMWH at weight-adjusted doses in most patients, without the need for laboratory monitoring. On the other hand, although treatment with UFH needs laboratory monitoring with Activated Partial Thromboplastin Time (aPTT), because its anticoagulant effect is unpredictable, it has the advantage that bleeding complications can be more easily managed, because UFH has a shorter half-life than LMWH, and can be effectively antagonized by protamine [1].

The antithrombotic efficacy of LMWH and UFH in the treatment of VTE and ACS has been evaluated in many randomized clinical trials and analyzed in several meta-analyses. Treatment of VTE with LMWHs is associated with similar or lower rates of recurrences and death as compared to treatment with UFH [2]; [3]. However, there is no evidence that LMWH are more effective than UFH in patients with ACS [4].

Minimizing the bleeding risk in patients treated with anticoagulants is of utmost clinical relevance, considering that major bleeding, anemia and blood transfusion are powerful and independent predictors of morbidity and mortality in patients with VTE or ACS on treatment with antithrombotic drugs [5]–[7]. To date, the systematic reviews comparing LMWH and UFH, focused on drug efficacy as primary end point and considered the incidence of bleeding a secondary end point. This choice could have affected the selection of studies to be included in the analysis, since some studies reporting haemorrhagic events could have been excluded due to the absence of the primary end point considered as inclusion criteria in those meta-analysis.

It has not been established yet whether the incidence of bleeding complications differs between LMWH and UFH.

Aim of our study was to perform a systematic review of randomized clinical trials to compare the incidence of major bleeding associated with the use of subcutaneous LMWH and intravenous UFH for treatment of acute VTE or ACS.

## Materials and Methods

### Data Sources and Searches

We attempted to identify all relevant published randomized controlled trials (RCT) that compared fixed-dose subcutaneous LMWH with adjusted-dose intravenous UFH in the initial treatment of thrombotic episodes. We searched MEDLINE, EMBASE and the Cochrane Central Register of Controlled Trials, using the search terms “randomized controlled trials” and “heparin” in combination with generic and trade names of individual preparations of LMWH. The search was completed in May 2010. We manually searched the references of retrieved publications to look for additional studies. No language restrictions were applied. Two investigators (EC, AMR) independently evaluated the studies for inclusion, and disagreements were resolved by discussion.

### Study Selection

In order to be included in this systematic review, published studies had to meet the following criteria: 1) study design: randomized controlled trial; 2) intervention: comparison of subcutaneous weight-adjusted, fixed-dose LMWH with adjusted doses (based on APTT values) intravenous UFH, for the initial treatment of acute thrombotic episodes; 3) availability of outcome data on the incidence of major bleeding. We accepted the definitions of major bleeding that were chosen in each individual trial. Dose-finding studies were excluded. Studies with no events in both LMWH and UFH arms were included in the descriptive analysis, but excluded from the meta-analysis, because calculation of the odds ratio (OR) was not feasible.

Pre-defined subgroup analyses were performed on the basis of the type of disease that was treated (VTE vs ACS), the doses of LMWH that were administered, the number of daily administrations of LMWH (one or two) and the type of LMWH used.

### Quality Assessment

In order to evaluate the quality of the included studies, we used the study-quality criteria of Jadad, which take into account proper randomization, allocation of patients and blinding [8].

### Data Extraction

Two investigators (EC, AMR) independently extracted the data on study design, study quality, incidence of major bleeding, patients’ characteristics, type, dose and number of daily administrations of LMWH, co-treatments, study end points and efficacy outcome. The data extracted for each trial were confirmed by consensus between the reviewers.

### Data Analysis

For each primary study that was included in the meta-analysis, a two-by-two table was constructed and the OR, with their 95% confidence intervals (CI), were calculated to estimate the risk of major bleeding in patients treated with LMWH compared to patients treated with UFH.

Pooled OR and 95% CI were calculated using the random effects model [9]. The homogeneity of the estimates of OR among studies was evaluated using the chi-square statistic test. Given the known difference of clinical settings of primary studies included in our meta-analysis (clinical heterogeneity), random effects analysis was performed for all studies, irrespective of the statistical significance of the heterogeneity chi-square test.

All analyses were performed using STATA 11.0 software [10].

## Results

### Study Selection

**Figure 1:**
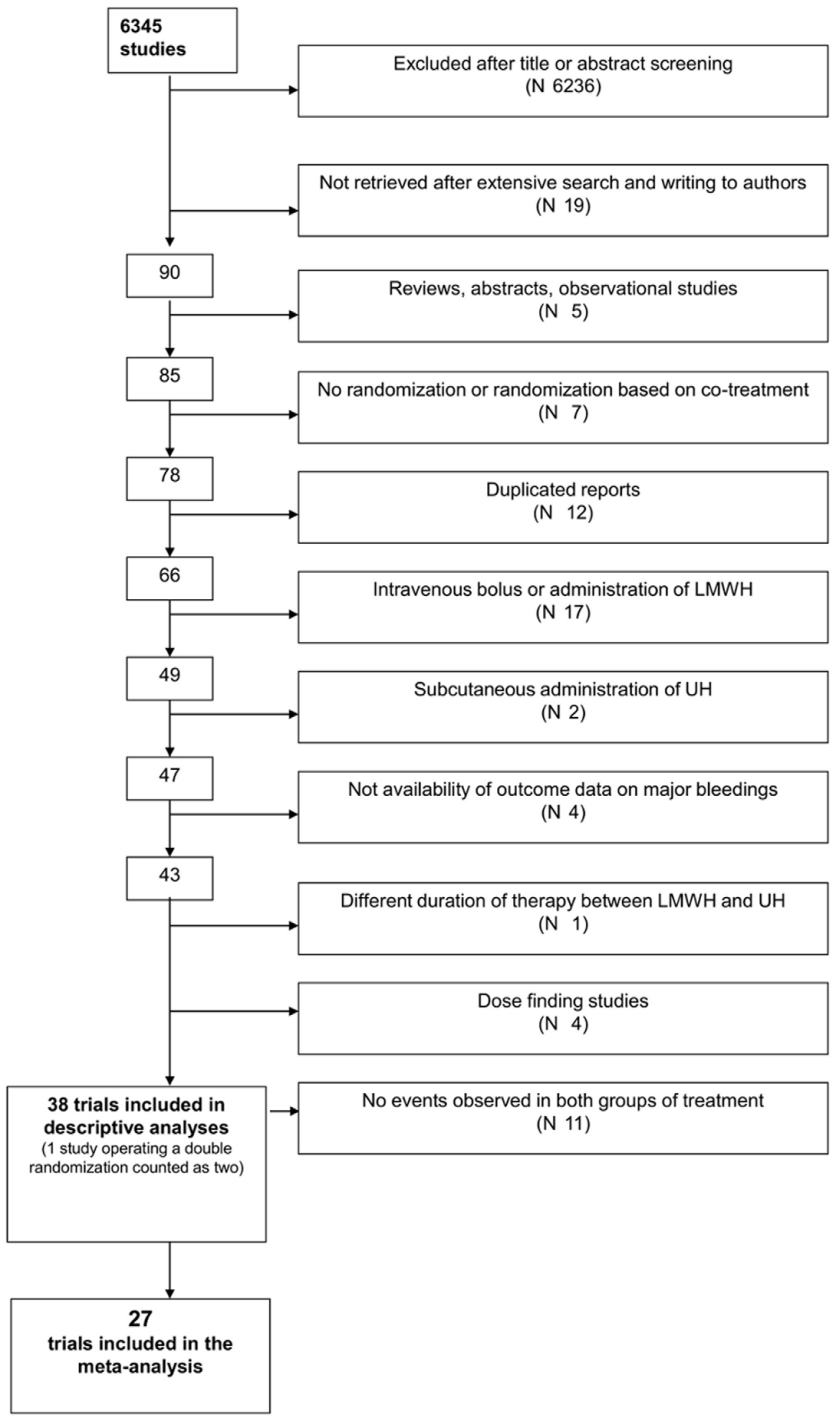
Study selection progression.

Figure 1 shows the process of study selection. We identified 6513 articles (1498 from Medline, 3205 from Embase, 1810 from the Cochrane Library). Of these, 1515 studies were duplicates, which were therefore eliminated, leaving 4998 articles for consideration. After the exclusion of irrelevant studies, which were identified by reviewing the titles and abstracts of all retrieved articles, 112 publications remained for analysis. We could not obtain the full publication of 19 studies after searching in the British Library and writing to the authors. Fifty-four of the remaining 93 articles were subsequently excluded, based on a more detailed evaluation of the full publications (Fig. 1). No additional studies were identified by reviewing the references from the original studies and other meta-analyses. One study, which operated a double randomization was considered as two data sets [11]. Thus, 37 studies were included in the descriptive analysis [11]–[47]. In 10 of them, no bleeding events were observed in any treatment group: therefore, 27 studies were included in the meta-analysis [12]–[15]; [17]–[38]; [47].

Table 1 describes the characteristics of the included studies. Thirty-four studies were published in English, one in French and two in Spanish, in journals of Internal Medicine, Cardiology, Hematology, or, less frequently, Angiology and Pneumology. The years of publication ranged between 1989 and 2006. Twenty-six studies enrolled patients with VTE, 11 studies enrolled patients with ACS (STEMI, NSTEMI, unstable angina). Nine different types of LMWH were used: the most commonly used one was enoxaparin. In all studies, the dose of intravenous UFH was titrated to maintain the APTT ratio between 1.5 and 2.5. The daily doses of subcutaneous LMWH ranged between 90 and 300 anti-Xa U/kg (mean 195 U/kg), in one or two daily administrations (9 and 28 studies respectively); one study allowed both the once daily and the twice daily administration [30]. Treatments lasted between 2 and 28 days (mean, 8 days). Concomitant medications for patients with ACS included Aspirin, Aspirin plus a thienopiridine, Aspirin plus a GPIIb-IIIa inhibitor, or Aspirin plus a thrombolytic agent. Most studies used the TIMI criteria to define major bleeding [48].

**Table 1:** Characteristics of included studies.

	Clinical indication	Patients n.		Major bleeding n. (%)	Mean age (years)	Type and daily dosage of LMWH	Cotreatments
Riess H et al. 2003	VTE	LMWH	627	6 (1.0)	61	Certoparin, 8000 IU, bid	None
		UFH	593	7 (1.3)			
Decousus et al. 1998	VTE	LMWH	195	7 (3.6)	72	Enoxaparin, 100 IU/Kg, bid	None
		UFH	205	8 (3.9)			
Columbus investigators 1997	VTE	LMWH	510	10 (2.0)	60	Reviparin, 3500–6300 IU, bid	None
		UFH	511	8 (1.6)			
Zhang Wang et al. 2006	ACS	LMWH	96	1 (1.0)	66	Parnaparin, 4250 IU, bid	ASA, UK
		UFH	90	3 (3.1)			
Hull et al. 1992	VTE	LMWH	213	1 (0.5)	No data	Tinzaparin, 175 IU/Kg, qd	None
		UFH	219	11 (5.0)			
Collaborative European Multicentre Study 1991	VTE	LMWH	85	2 (2.4)	No data	Nadroparin, 12500–17500 IU, bid	None
		UFH	81	4 (4.9)			
Campos et a. 2002	ACS	LMWH	107	1 (0.9)	60	Enoxaparin, 80 IU/Kg, bid	ASA
		UFH	96	9 (9.4)			
Goldhaber et al. 1998	VTE	LMWH	41	1 (2.4)	54	Ardeparin, 130 /Kg, bid	None
		UFH	39	1 (2.6)			
Simonneau et al. 1997	VTE	LMWH	304	3 (1.0)	67	Tinzaparin, 175 IU/Kg, qd	None
		UFH	308	5 (1.6)			
PRIME CARE Study Investigators Group 2005	ACS	LMWH	451	2 (0.4)	57	Parnaparin, 6400 IU, qd	ASA
		UFH	446	2 (0.4)			
Goodman et al. 2003	ACS	LMWH	380	8 (2.1)	64	Enoxaparin, 100 IU/Kg, bid	ASA, EPF
		UFH	366	20 (5.5)			
Kakkar et al. 2003	VTE	LMWH	126	0 (0)	No data	Bemiparin, 115 IU/Kg, qd	None
		UFH	126	1 (1.0)			
Prandoni et al. 1992	VTE	LMWH	85	1 (1.2)	No data	Nadroparin, 12500–17500 IU, bid	None
		UFH	85	3 (3.5)			
Levine et al. 1996	VTE	LMWH	247	5 (2.0)	58	Enoxaparin, 100 IU/Kg, bid	None
		UFH	253	3 (1.2)			
Gurfinkel et al. 1995	ACS	LMWH	68	0 (0)	63	Nadroparin, 214 IU/Kg, bid	ASA
		UFH	70	2 (2.9)			
Harenberg et al. 2000	VTE	LMWH	265	4 (1.5)	62	Certoparin, 8000 IU, bid	None
		UFH	273	11 (4.0)			
Cohen et al. 2002	ACS	LMWH	315	4 (1.3)	64	Enoxaparin, 100 IU/Kg, bid	ASA, TFB
		UFH	210	3 (1.4)			
Blazing et al. 2004	ACS	LMWH	2026	18 (0.9)	61	Enoxaparin, 100 IU/Kg, bid	ASA, TFB
		UFH	1961	8 (0.4)			
Breddin et al. 2001	VTE	LMWH	762	2 (0.3)	59	Reviparin, 7000–12600 IU, qd or bid	None
		UFH	375	2 (0.6)			
Perez de Llano et al. 2003	VTE	LMWH	29	1 (3.4)	No data	Enoxaparin, 100 IU/Kg, bid	None
		UFH	21	0 (0)			
Fiessinger et al. 1996	VTE	LMWH	120	0 (0)	61	Dalteparin, subcutaneous bolus 5000 IU, then 200 IU/kg qd	None
		UFH	133	2 (1.5)			
Harenberg et al. 1990	VTE	LMWH	24	3 (12.5)	61	Certoparin, 150 IU/Kg, bid	None
		UFH	26	3 (11.5)			
Luomanmaki et al. 1996	VTE	LMWH	117	0 (0)	59	Dalteparin, subcutaneous bolus 5000 IU, then 200 IU/kg qd	None
		UFH	131	1 (0.8)			
Koopman et al. 1996	VTE	LMWH	202	1 (0.5)	60	Nadroparin, 8200–18400 IU, bid	None
		UFH	198	2 (1.0)			
Cohen et al. 1997	ACS	LMWH	1607	102 (6.3)	63	Enoxaparin, 100 IU/Kg, bid	ASA
		UFH	1564	107 (6.8)			
SYNERGY Trial Investigators 2004	ACS	LMWH	4993	453 (9.1)	68	Enoxaparin, 100 IU/Kg, bid	ASA clopidogrel/antiIIb/IIIa
		UFH	4985	379 (7.6)			
Kirchmaier et al. 1998	VTE	LMWH	128	1 (0.8)	61	Certoparin, 8000 IU, bid	None
		UFH	131	4 (3.1)			
Belcaro et al. 1999	VTE	LMWH	98	0 (0)	53	Nadroparin, 100 IU/Kg, bid	None
		UFH	97	0 (0)			
Malhotra et al. 2001	ACS	LMWH	51	0 (0)	60	Enoxaparin, 100 IU/Kg, bid	ASA
		UFH	42	0 (0)			
Moreno Palomares et al. 2001	VTE	LMWH	17	0 (0)	67	Dalteparin, 200 IU/Kg qd	None
		UFH	15	0 (0)			
Simonneau et al. 1993	VTE	LMWH	67	0 (0)	62	Enoxaparin, 100 IU/Kg, bid	None
		UFH	67	0 (0)			
Meyer et al. 1995	VTE	LMWH	29	0 (0)	61	Dalteparin, 120 IU/kg, bid	None
		UFH	31	0 (0)			
Findik et al. 2002	VTE	LMWH	29	0 (0)	50	Enoxaparin, 100 IU/Kg bid	None
		UFH	30	0 (0)			
Lindmarker et al. 1994	VTE	LMWH	101	0 (0)	61	Dalteparin, 200 IU/Kg, qd	None
		UFH	103	0 (0)			
Stricker et al. 1999	VTE	LMWH	9	0 (0)	66	Nadroparin, 185 IU/Kg, qd	None
		UFH	11	0 (0			
Kim et al. (a) 2005	ACS	LMWH	40	0 (0)	63	Dalteparin, 120 IU/Kg, bid	ASA, Clopidogrel
		UFH	40	0 (0)			
Kim et al. (b) 2005	ACS	LMWH	40	0 (0)	59	Dalteparin, 120 IU/Kg, bid	ASA, Clopidogrel, TFB
		UFH	40	0 (0)			
Aiach et al. 1989	VTE	LMWH	31	0 (0)	62	Dalteparin, 100 IU/Kg bid, then in function of antifactor Xa, bid	None
		UFH	30	0 (0)			

**n**: numbers; **LMWH**: low molecular weight heparin; **UFH**: unfractioned heparin; **VTE**: venous thromboembolism; **ACS**: acute coronary syndrome; **qd**: once daily; **bid**: twice daily; **tid**: three times a day; **UK**: urochinasi; **TFB**: tirofiban; **EPF**: eptifibatide; **antiIIb/IIIa**: GPIIb/IIIa inhibitors; **ASA**: acetylsalicylic acid.

A total of 28,637 patients had been enrolled in the studies, 14,635 of whom were treated with LMWH and 14,002 with UFH. The mean number of patients enrolled in each trial was 754 (range 20–9,978 ), their mean age was 62 y (range 49–73), and 63% (range 31–83%) were men.

The aim of the majority of the studies was to evaluate combined end-points; less frequently, a single clinical end-point or an instrumental measure were evaluated. Major bleeding ranged from 0 to 12.5%, (mean, 4.4%) in patients treated with LMWH and from 0 to 11.5% (mean, 4.4%) in patients treated with UFH. The mean incidence of bleeding events were lower in VTE (1.1% with LMWH and 1.9% with UFH), compared to ACS (5.8% with LMWH and 5.4% with UFH). Considering the efficacy end-points, 15 studies showed that LMWH were superior to UFH [12]; [15]; [17]–[19]; [21]–[23]; [26]; [27]; [29]; [30]; [36]; [40]; [42], 1 study showed that UFH was superior to LMWH [46], while the remaining 21 showed that there was no statistically significant difference between the two treatments [11]; [13]; [14]; [16]; [20]; [24]; [25]; [28]; [31]–[35]; [37]–[39]; [41]; [43]–[45]; [47].

### Assessment of Study Quality

Based on the study-quality criteria of Jadad [8], 3 studies scored 1 point, 17 scored 2 points, 14 scored 3 points, 1 scored 4 points and 2 scored 5 points. Lack of blindness was the most common flaw in the studies with a low Jadad score.

### Data Synthesis

**Figure 2:**
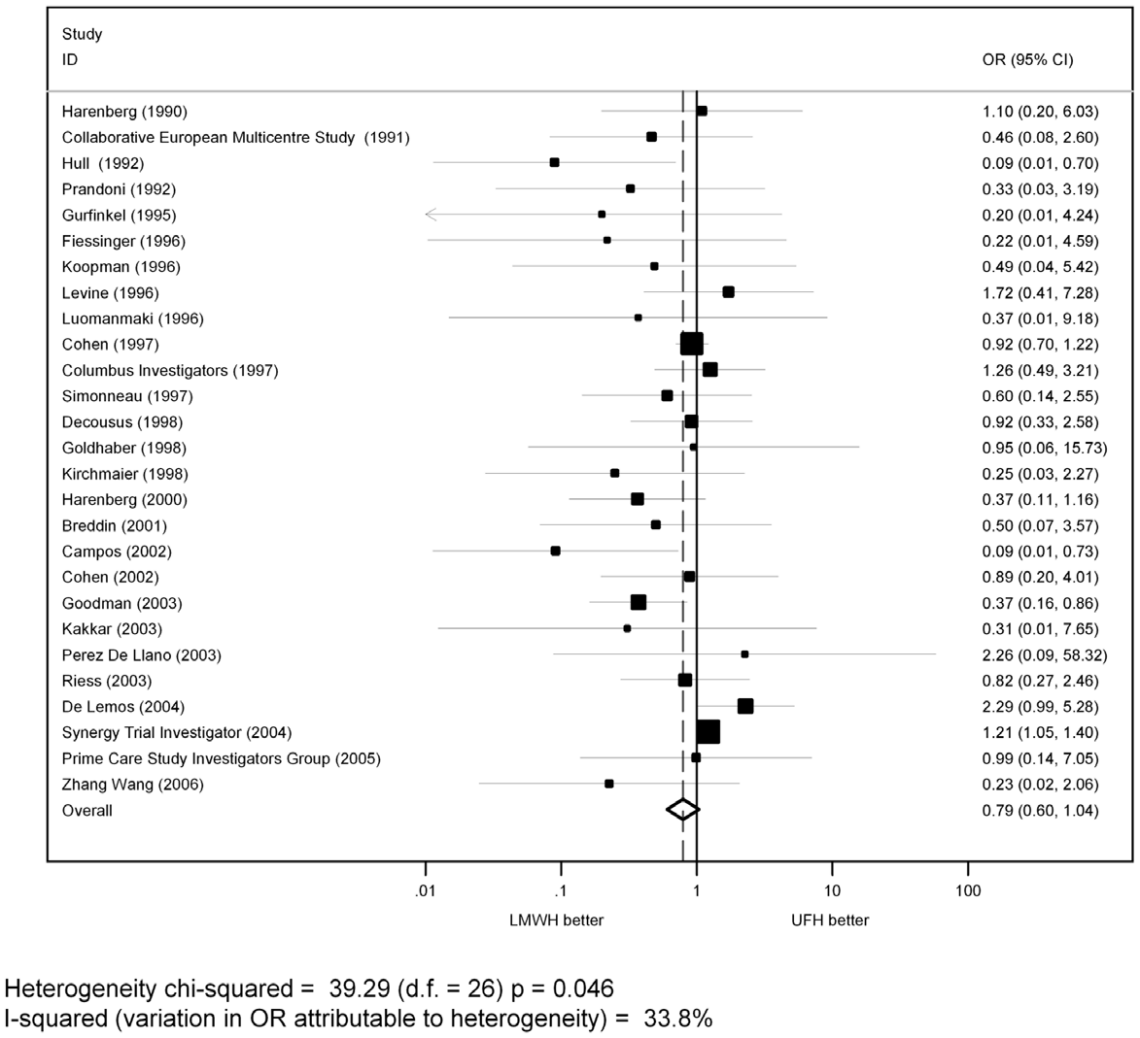
Pooled estimates of OR for major bleedings of LMWH versus UFH in all patients.

Pooled estimates of ORs for major bleeding showed a non-statistically significant trend in favor of LMWH compared to UFH (OR = 0.79, 95% CI: 0.60–1.04, p = 0.091; n = 27 primary studies) (Figure 2).

**Figure 3:**
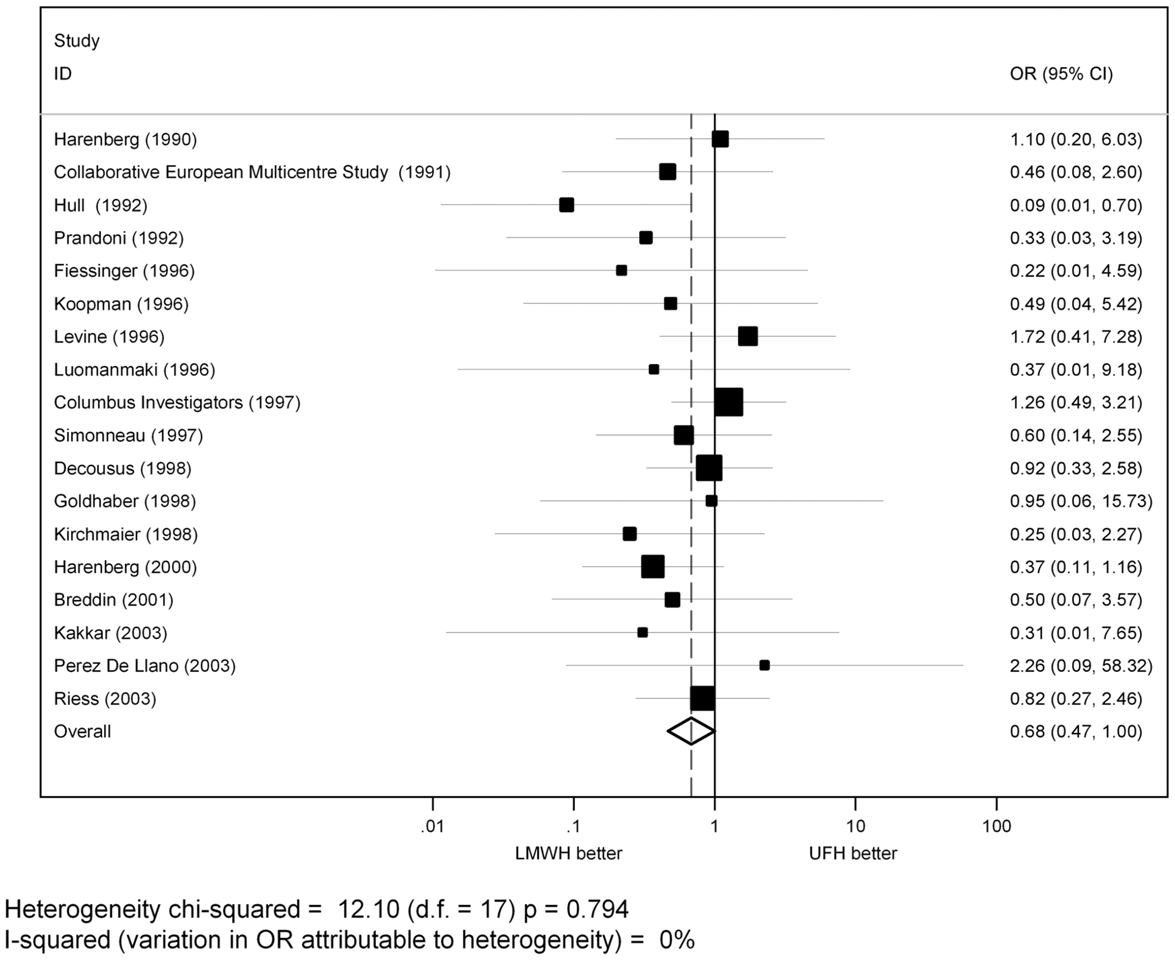
Pooled estimates of OR for major bleedings of LMWH versus UFH in VTE patients.

When the analysis was limited to trials that enrolled patients with VTE, pooled estimates of OR was in favor of LMWH (OR = 0.68, 95% CI: 0.47–1.00, p = 0.05) (figure 3).

### Study Characteristics

**Figure 4:**
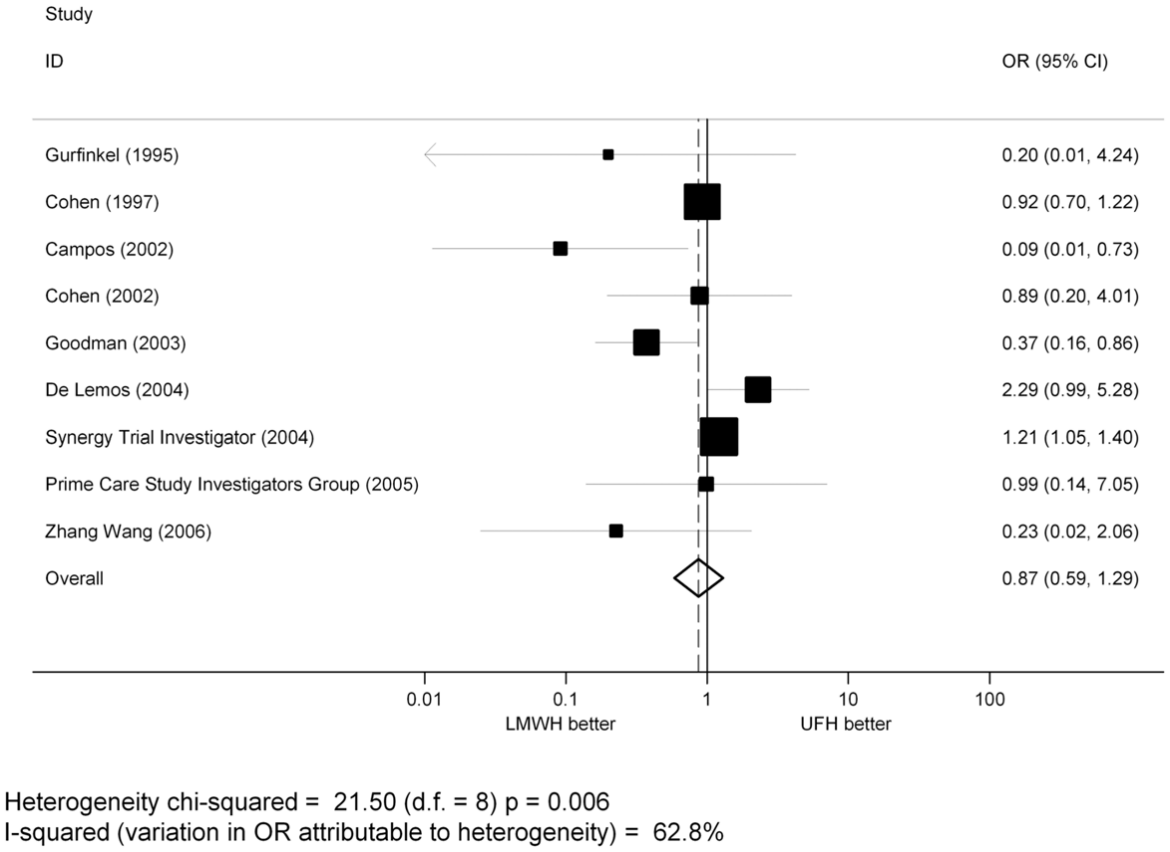
Pooled estimates of OR for major bleedings of LMWH versus UFH in ACS patients.

In contrast, when the analysis was limited to trials that enrolled patients with ACS, no statistically significant differences between LMWH and UFH were found (OR = 0.87, 95% CI: 0.59–1.29; p = 0.493) (figure 4).

Once daily LMWH was significantly safer than UFH, (OR = 0.39, 95% CI: 0.16–0.95, p = 0.039), while, for twice daily LMWH, no statistical difference was observed compared to UFH (OR = 0.86, 95% CI: 0.65–1.14, p = 0.296) (figure S1). When the analysis was limited to the subgroup of VTE trials, once daily LMWH was significantly safer than UFH (OR = 0.31, 95% CI: 0.12–0.84), while no significant differences were found when considering the other subgroups (VTE twice, ACS once and ACS twice) (figure S2).

The exclusion of studies in which LMWH was under-dosed (less than 75% of the recommended daily dose) or overdosed (more than 125% of the recommended dose) did not substantially change the results (OR = 0.79, CI 95% 0.58–1.06, p = 0.121) (figure S3).

No statistically significant differences among different LMWH were observed (figure S4).

The results did not change, after Exclusion of the low quality studies (Jadad score <3) from the analysis (OR = 0.88, 95% CI: 0.67–1.15, p = 0.342) (figure S5).

Based on our a priori defined protocol, we excluded dose-finding studies [49]–[52], which had been included in other revisions. In particular, in the VTE setting, the study by Merli et al., which used two different LMWH doses, compared to a single UFH group, was excluded despite the large number of patients enrolled. For the sake of completeness, we repeated our analysis including the dose-finding trials and the results did not change (figure S6).

## Discussion

Our systematic review shows a trend toward a non-statistically significant lower incidence of major bleeding with LMWH compared to UFH (OR 0.79), in the treatment of acute thrombotic events, such as VTE and ACS. When we analyzed separately the RCT that enrolled VTE patients and those enrolling ACS patients, the reduction in the bleeding risk associated with LMWH (OR = 0.68, p = 0.05) reached statistical significance for VTE. Some reasons might explain the absence of significant difference in bleeding between LMWH and UFH in ACS patients: i) many ACS patients underwent invasive procedures (coronary angiography and PCI), ii) ACS patients were often in co-treatment with antiplatelet agents; iii) LMWH patients are likely to be more often in the therapeutic range compared to dose adjusted UFH.

Somewhat unexpectedly, the incidence of bleeding complications was significantly lower with once daily administrations of LMWH, compared to twice daily administrations, the total daily doses being similar in the two treatment regimens (figure S1). Although we have not a clear explanation of these results, it could be speculated that bleeding correlates better with the trough drug concentrations (lower in once a day than in twice a day administrations) than with peak concentrations (higher in daily administration).

Anyway, our findings raise the possibility that dosing regimens can be important determinants for bleeding complications. Further studies should clarify this topic.

The use of LMWH and UFH for treatment of VTE and ACS has been evaluated in previous systematic reviews and meta-analyses, which were focused on their antithrombotic efficacy as primary end-point. However, in consideration of the negative impact on mortality and adverse cardiovascular events associated with major bleeding [5]–[7], it is also important to establish which treatment is safer, in terms of incidence of bleeding complications.

As far as treatment of VTE is concerned, only the meta-analysis of Lim et al on LMWH-associated bleeding is available to date [53], which demonstrated a higher incidence of bleeding complications in patients with renal failure, without comparing LMWH with UFH. Information on the difference in bleeding complications between LMWH and UH is retrievable from two other meta-analyses, which were mainly focused on evaluating the differences in efficacy between the two treatments [2]; [3]. Quinlan et at. reported a non-statistically significant trend toward lower frequency of major bleeding associated with LMWH, compared to UFH (1.4% vs 2.3%, OR 0.67, 95% CI 0.36–1.27), in patients with non-massive pulmonary embolism [2]. In the Systematic Review of the Cochrane Collaboration on VTE [3], LMWH were shown to be significantly safer than UFH (incidence of major bleeding, 1% vs 2.1%, OR 0.57; 95% CI 0.39–0.83). While, at a first glance, our review seems to be very similar to the Cochrane review, we think that they are basically different. The recently published Cochrane meta-analysis focused on the comparison between LMWH and unfractionated heparin (UFH in terms of their efficacy for the initial treatment of venous thromboembolism (VTE). In our manuscript we have chosen the safety of these drugs for the initial treatment of acute thrombotic events as primary end point. As a consequence, in our review all the studies reporting haemorrhagic events, irrespective of the efficacy endpoint that was reported, have been included. As a consequence, compared to the Cochrane meta-analysis, 9 additional studies were included in our descriptive analysis [19]; [23]; [30]; [31]; [33]; [41]; [43]; [46]; [47], 6 of which were also included in our meta-analysis [31]; [33]; [41]; [43]; [46]; [47]. These additional studies account for 1344 more VTE patients included in our analysis.

As far as ACS treatment is concerned, three meta-analysis have recently been published, which gave contrasting results. Magee et al [54], consistently with our data, showed a similar rate of major bleeding in LMWH and UFH-treated patients with ACS (RR = 1), despite the fact that only one of the 7 studies included by Magee met our inclusion criteria. In contrast, Murphy et al [55], who analyzed studies comparing enoxaparin and UFH only, found an excess of major bleeding in the enoxaparin group (OR 1.25, p = 0.019). We have no explanation for the contrasting results of the analysis by Murphy et al, compared to those of the study by Magee et al and of our systematic review. The fact that Murphy et al [55] focused on studies that used enoxaparin only does not apparently account for the differences in results, because, when we restricted our analysis to studies that used enoxaparin only, we found no statistically significant differences in the incidence of major bleeding between the enoxaparin group and the UFH group (figure S4). The inclusion of studies that administered enoxaparin either intravenously or subcutaneously in the meta-analysis by Murphy et al might account for the different results obtained in our meta-analysis, which included those studies that administered LMWHs subcutaneously only. This concept seem to be strengthened by the most recent meta-analysis by Silvain et al [56], that enrolled RCT’s and registers studies and included only patients treated with enoxeparin during percutaneous coronary interventions. While they find an important reduction in major bleeding in patients treated with intravenous Enoxeparin, the risk of bleeding using Enoxeparin by the subcutaneous route was the same as UFH.

The results of our study may have important clinical implications. Patients at high risk for thrombotic events are often also at high risk for bleeding [57]; [58]. Iron deficiency anemia and/or hemorrhagic diathesis are common co-morbidities. In this context, the choice of the best anticoagulant treatment should be done taking into account the risk of adverse events more than the therapeutic efficacy. As a matter of fact, minimizing the bleeding risk in patients treated with anticoagulants is of utmost clinical relevance, considering that major bleeding, anemia and blood transfusion are powerful and independent predictors of morbidity and mortality in patients with VTE or ACS on treatment with antithrombotic drugs [5]–[7]. LMWH has the advantages of subcutaneous administration, more predictable anticoagulant response, lack of the need for laboratory monitoring and probably less risk of major bleeding in the VTE. On the other hand the lack of complete antagonization by antidotes and the long acting profile can be a disadvantage in active bleeding patients. UFH is less easy to handle, but the presence of an antidote and its brief half life could be arguments to consider in choosing the type of heparin to use in high bleeding risk patients who need anticoagulation. The choice must be based on the single patient and on the clinical and practical context, taking into account the bleeding profile of different heparins in different settings.

### Limitations

The major limitation of our systematic review is the clinical heterogeneity between studies, especially considering all the trials together. Sources of clinical heterogeneity are several.

The primary outcome of our systematic review, major bleeding, was the secondary end-point of the original RCT that we considered in our analysis. As we included all the RCT that compared subcutaneous LMWH with UFH, we considered different clinical scenarios and consequently different study designs, in which heparins were used at different doses and with different co-therapies. However, our results should not be affected by these factors, since we considered randomized studies only. Moreover, trying to take into account clinical heterogeneity, we used a random effect model for the analysis, which is known to be more conservative.

We chose the criteria that had been used in the original studies as criteria for major bleeding and this could account for some of the heterogeneity present in our results. However, most studies considered the following major bleeding events, which are undoubtedly clinically relevant: i) the presence of intracranial or retroperitoneal haemorrhage, ii) haemorrhage that led directly to death, necessitated transfusion or led to the interruption of antithrombotic treatment, iii) a ≥2 g/dl decrease in the haemoglobin concentration.

Finally, in the arterial setting clinical and statistical heterogeneity does not allow to derive definitive conclusions; vice versa, in venous setting the sources of clinical heterogeneity are lower as confirmed by the absence of statistical heterogeneity.

In conclusion, the results of our systematic review suggest that LMWH might have a better safety profile than UFH in the treatment of VTE, while no differences between the two treatments was detected in ACS. The choice of which heparin to use to minimize bleeding risk must be based on the single patient and on the clinical and practical context, taking into account the bleeding profile of different heparins in different settings.

## Supporting Information

Figure S1 **Subgroup analysis: number of daily LMWH administrations (once daily vs twice daily).**
(DOC)

Figure S2 **Subgroup analysis: number of daily LMWH administrations (once daily vs twice daily) in VTE and ACS studies.**
(DOC)

Figure S3 **Subgroup analysis, after the exclusion of studies in which LMWH was was under-dosed (less than 75% of the recommended daily dose) or overdosed (more than 125% of the recommended dose).**
(DOC)

Figure S4 **Subgroup analysis by type of LMWH.**
(DOC)

Figure S5 **Subgroup analysis, after exclusion of low quality studies (Jadad Score<3).**
(DOC)

Figure S6 **Overall meta-analysis, including dose-finding studies (studies grouped by VTE or ACS patients).**
(DOC)
