# Supplementary material for: Bleeding Risk during Treatment of Acute Thrombotic Events with Subcutaneous LMWH Compared to Intravenous Unfractionated Heparin; A Systematic Review
Source: PLoS One. 2012 Sep 11;7(9):e44553. doi: 10.1371/journal.pone.0044553 (PMC3439371; doi:10.1371/journal.pone.0044553)
Supplement: Figure S3 — Subgroup analysis, after the exclusion of studies in which LMWH was was under-dosed (less than 75% of the recommended daily dose) or overdosed (more than 125% of the recommended dose). (DOC) [file pone.0044553.s003.doc]

**Figure S 3**
