# Supplementary material for: Bleeding Risk during Treatment of Acute Thrombotic Events with Subcutaneous LMWH Compared to Intravenous Unfractionated Heparin; A Systematic Review
Source: PLoS One. 2012 Sep 11;7(9):e44553. doi: 10.1371/journal.pone.0044553 (PMC3439371; doi:10.1371/journal.pone.0044553)
Supplement: Figure S5 — Subgroup analysis, after exclusion of low quality studies (Jadad Score<3). (DOC) [file pone.0044553.s005.doc]

**Figure S 5**
